# Distinct expression of functionally glycosylated alpha-dystroglycan in muscle and non-muscle tissues of FKRP mutant mice

**DOI:** 10.1371/journal.pone.0191016

**Published:** 2018-01-10

**Authors:** Anthony Blaeser, Hiroyuki Awano, Pei Lu, Qi-Long Lu

**Affiliations:** McColl-Lockwood Laboratory for Muscular Dystrophy Research, Carolinas HealthCare System, Charlotte, North Carolina, United States of America; Rutgers University Newark, UNITED STATES

## Abstract

The glycosylation of alpha-dystroglycan (α-DG) is crucial in maintaining muscle cell membrane integrity. Dystroglycanopathies are identified by the loss of this glycosylation leading to a breakdown of muscle cell membrane integrity and eventual degeneration. However, a small portion of fibers expressing functionally glycosylated α-DG (F-α-DG) (revertant fibers, RF) have been identified. These fibers are generally small in size, centrally nucleated and linked to regenerating fibers. Examination of different muscles have shown various levels of RFs but it is unclear the extent of which they are present. Here we do a body-wide examination of muscles from the FKRP-P448L mutant mouse for the prevalence of RFs. We have identified great variation in the distribution of RF in different muscles and tissues. Triceps shows a large increase in RFs and together with centrally nucleated fibers whereas the pectoralis shows a reduction in revertant but increase in centrally nucleated fibers from 6 weeks to 6 months of age. We have also identified that the sciatic nerve with near normal levels of F-α-DG in the P448Lneo- mouse with reduced levels in the P448Lneo+ and absent in LARGE^myd^. The salivary gland of LARGE^myd^ mice expresses high levels of F-α-DG. Interestingly the same glands in the P448Lneo-and to a lesser degree in P448Lneo+ also maintain considerable amount of F-α-DG, indicating the non-proliferating epithelial cells have a molecular setting permitting glycosylation.

## Introduction

Dystroglycanopathies are characterized by a common secondary defect in dystroglycan (DG) glycosylation. The defect is related to autosomal-recessive mutations in at least 18 different genes including *fukutin-related protein* (*FKRP*), *fukutin*, *LARGE*, *POMGnT1*, *POMT1*, *POMT2*, *Isoprenoid Synthase Domain Containing (ISPD)*, *Transmembrane protein 5 (TMEM5)*, *β1*,*3-N-acetylglucosaminyltransferase1 (B3GNT1)*, *glycosyltransferase-like domain containing 2 (GTDC2)*, *β3-N-acetylgalactosaminyltransferase 2 (B3GALNT1)*, *and SGK196* [1–7]. These have been suggested to be involved in post-translational glycosylation modifications of α-DG [8–14],crucial in maintaining the connection with the extracellular matrix and muscle membrane integrity. This is done through the binding of the glycan to various extracellular matrix (ECM) proteins such as agrin, perlecan, neurexin and pikachurin [15–21]. In dystroglycanopathies, the presence of glycosylated α-DG is significantly reduced or absent leading to the breakdown of muscle membrane integrity and eventual degeneration. However, a small subset of muscle fibers can be seen with near normal levels of glycosylated α-DG, referred to as revertant fibers (RF).

The functionally glycosylated α-DG (F-α-DG) has been typically demonstrated by specific antibodies recognizing the sugar epitope of the protein, the most widely used being monoclonal antibodies IIH6 and VIA4. In contrast to the strong membrane signal with the antibodies to the F-α-DG in all fibers of normal skeletal and cardiac muscles, the majority of fibers in dystroglycanopathy muscles lack detectable F-α–DG in patients with *FKRP* mutations [22]. However, a small number of RFs have been reported in muscle biopsy samples of patients with *FKRP* mutations. Similarly, the RF has now been demonstrated in *FKRP* mutant animals. Our earlier study showed that RFs are present in limb muscles, diaphragm and occasionally in cardiac muscles [23]. Furthermore, we also demonstrated that restoration of F-α-DG in the *FKRP* mutant mice is associated with muscle regeneration and correlated to the markers of muscle maturation [24]. Investigation in the new born FKRP-P448Lneo- mutant mice reveals near normal levels of F-α-DG expressed in skeletal muscles within a few days after birth with decreasing levels and expression becoming barely detectable by postnatal day 7. Levels of F-α-DG expression is much lower and detectable only for the first 4 days in cardiac muscle. No F-α-DG is observed in all muscles of the LARGE^myd^ mice. These data suggest that modulation of glycosylation of F-α-DG is tissue specific, developmentally regulated and associated with regeneration.

Mutations of *FKRP* affect organs and tissues differentially. Mild limb girdle muscular dystrophies are accompanied by little or no brain defects however the more severe forms, such as congenital muscular dystrophy, present severe brain and eye abnormalities. This wide range of clinical phenotypes has been similarly demonstrated in animals with glycosylation defects of α-DG as the consequence of *FKRP* mutations [25]. It has also been noted that mutations in different glycosyltransferases result in different severities of neuronal abnormalities. LARGE mutant mice present clear brain and nerve impairment at early stages of development [8] while P448Lneo- mice demonstrate little to no neuronal defects [25]. The mechanism(s) underlying these variations is still unclear Furthermore, different mutations may well affect the F-α-DG differently due to tissue specific pathways of glycosylation. One such example is the expression of F-α-DG in salivary gland of LARGE^myd^ mice. This has been attributed to the expression of LARGE-2, compensating for the defect in the *LARGE* gene [26]. These lines of evidence together suggest the probability of wide variations in expression of F-α-DG in different tissues temporarily or permanently of different genetic background including *FKRP* mutations.

Here we broaden our examination for F-α-DG to more skeletal muscles not previously covered as well as other tissues, body-wide, to elucidate the extent with which revertant fibers are present. We have identified muscles that are more severely affected by the loss of F-α-DG. However, severity in muscle regeneration is not always correlated with increased revertant fibers. Of interest is the expression of near normal levels of F-α-DG with laminin binding capacity in the peripheral nerve, salivary glands and large intestine of adult FKRP-P448Lneo- mutant mice. The pattern and distribution of glycosylation provide key insight into the possible mechanism(s) of glycosylation as well as targets for therapy.

## Materials and methods

### Animals and ethical statement

Mice containing a homozygous, missense mutation (*c*.*1343C>T*, p.Pro448Leu) in the *fukutin-related protein* (*FKRP)* gene were previously generated in the McColl Lockwood Laboratory for Muscular Dystrophy Research [25, 27]. These mice are available with the neomycin selection cassette present (P448Lneo+) or removed (P448Lneo-). LARGE^myd^ and C57Bl/6 mice were purchased from Jackson Laboratory (Bar Harbor, ME, USA) with LARGE^myd^ mice continually bred in house. This study was carried out in strict accordance with the recommendations in the Guide for the Care and Use of Laboratory Animals of the National Institutes of Health. The protocol was approved by the Committee on the Ethics of Animal Experiment of IACUC Carolinas Medical Center (01-13-04A). All efforts were made to minimize suffering. Three animals for each P448Lneo- (aged 6–8 weeks and 6 months), P448Lneo+, LARGE^myd^ and C57BL/6 (aged 6–8 weeks) were used.

### Histology and immunohistochemistry

Tissues were snap frozen in chilled isopentane. Cross sections of 6 μm thickness were cut from the frozen tissues. Immunohistochemical staining of functional glycosylation of α-DG was performed on cross sections of the muscle and non-muscle tissues fixed with ice-cold 50% ethanol and 50% acetic acid for 1 minute, and blocked with 8% bovine serum albumin (BSA) in ddH_2_O for 1 hour. Primary antibody against F-α-DG (IIH6C4; Millipore, Temecula, CA, USA) in 1% BSA at 1:500 dilution was incubated overnight at 4°C. Following primary antibody, sections were washed three times, for 10 minutes each, with PBS and incubated with Alexa Fluor 488-conjugated anti mouse (Life Technologies, Carlsbad, CA, USA) at 1:500 dilution. Sections were also stained with secondary antibody only as negative controls.

Immunofluorescence was visualized using an Olympus BX51 fluorescent microscope (Opelco, Dulles, VA, USA). Images were captured using an Olympus DP70 CCD camera system (Opelco, Dulles, VA, USA) at standard gain and same exposure time.

For fiber diameter, central nucleation and revertant fiber counts three separate images were taken for each muscle of interest with a total across all three sections used for each animal. A minimum of 300–500 muscle fibers for each muscle in each animal for a total of approximately 1000–1500 fibers were examined across three animals for each muscle at each age. Fiber diameter was determined by measuring the equivalent radius using MetaMorph (Molecular Devices LLC, Sunnyvale, CA, USA) and multiplying the radius by 2. Total centrally nucleated fibers are reported as a percentage of the total with SEM. Revertant fibers were counted among the same images with the same number of fibers. Fibers with strong IIH6 staining surrounding a majority of an individual fiber, clearly over background staining, was counted as revertant. Revertant fibers are expressed as a percentage of the total with SEM.

### Protein extraction and western blot

Total proteins were extracted from tissues using TX-100 buffer (1% Triton X-100, 50mM Tris pH8.0, 150mM NaCl, 0.1% SDS) supplemented with protease inhibitor cocktail (Roche, Germany). Samples were homogenized in TX-100 buffer and the supernatants were collected by centrifugation at 16,000*g* for 10 minutes. Protein concentration was determined by modified Lowry assay (Bio-Rad DC protein assay). The lysates were then loaded on 4–20% Tris-glycine gel (Invitrogen, Carlsbad, CA, USA). Protein was transferred to polyvinylidene difluoride (PVDF) membranes and incubated with protein-free T20 blocking buffer (Pierce, Rockford, IL). The membrane was incubated with antibodies against α-DG (IIH6C4) and α-actin (Sigma, St. Louis, MO, USA) in 20mM Tris pH7.4, 150mM NaCl, 0.1% Tween20 at 1:2000 dilutions. Alpha-DG and α-actin antibodies were detected by HRP-Goat anti-mouse IgM (Invitrogen, Carlsbad, CA, USA) and Goat anti-rabbit IgG-HRP conjugate (Bio-Rad, Hercules, CA, USA) respectively. Blots were developed with ECL (PerkinElmer, Waltham, MA, USA) and the images were exposed and processed by a LAS-4000 imaging system (Fujifilm, Valhalla, NY, USA).

#### RNA Extraction and Quantitative RT-PCR

Total RNA was extracted from tissues using TRIZOL® extraction method. Shavings were homogenized in 1mL of Trizol® followed by 200ul chloroform addition and shaken to mix. Samples were centrifuged, and aqueous phase removed to new tube. Equal volume (~500ul) of isopropanol was added and tubes inverted several times followed by a 10 minute room temperature incubation. Samples were centrifuged, and supernatant removed leaving the RNA pellet. RNA pellet was washed with 75% ethanol and allowed to air dry for 5–10 minutes. RNA was subsequently re-suspended in appropriate volume of water (~20ul). RNA concentration and O.D. was determined using the Nanodrop 2000c (ThermoFisher Scientific). Quantitative RT-PCR was performed on a BioRad C1000 system (BioRad) using Taqman gene expression assays (ThermoFisher Scientific) for LARGE (Mm00321885_m1), LARGE-2 (Gyltl-1b; Mm00554377_g1) and FKRP (Mm00557870_m1) with GAPDH (Mm99999915_g1) internal controls. The 2^ΔΔCt was calculated and expression levels expressed as relative expression to C57 control tissues.

### Laminin binding assay

Proteins were transferred to PVDF membranes and blocked for 1 hour at 4°C in laminin overlay buffer (10mM ethanolamine, 140mM NaCl, 1mM MgCl_2_ and 1mM CaCl_2_, pH 7.4) containing 5% nonfat dry milk. Membrane was incubated with laminin from Engelbreth-Holm-Swarm murine sarcoma basement membrane (Sigma, St. Louis, MO, USA) overnight at 4°C. Anti-laminin antibody (Sigma, St. Louis, MO, USA) was incubated at 1:1500 dilutions. Antibody against laminin was detected by Goat anti-rabbit IgG-HRP conjugate (Bio-Rad, Hercules, CA, USA) and blots were developed with ECL. Images were exposed and processed by a LAS-4000 imaging system (Fujifilm, Valhalla, NY, USA).

### Statistics

Statistical analysis was performed using Excel 2010 and an unpaired t-test with a statistical significance achieved with p<0.05.

## Results

### Revertant fibers in skeletal muscles and association with regeneration

**Fig 1:**
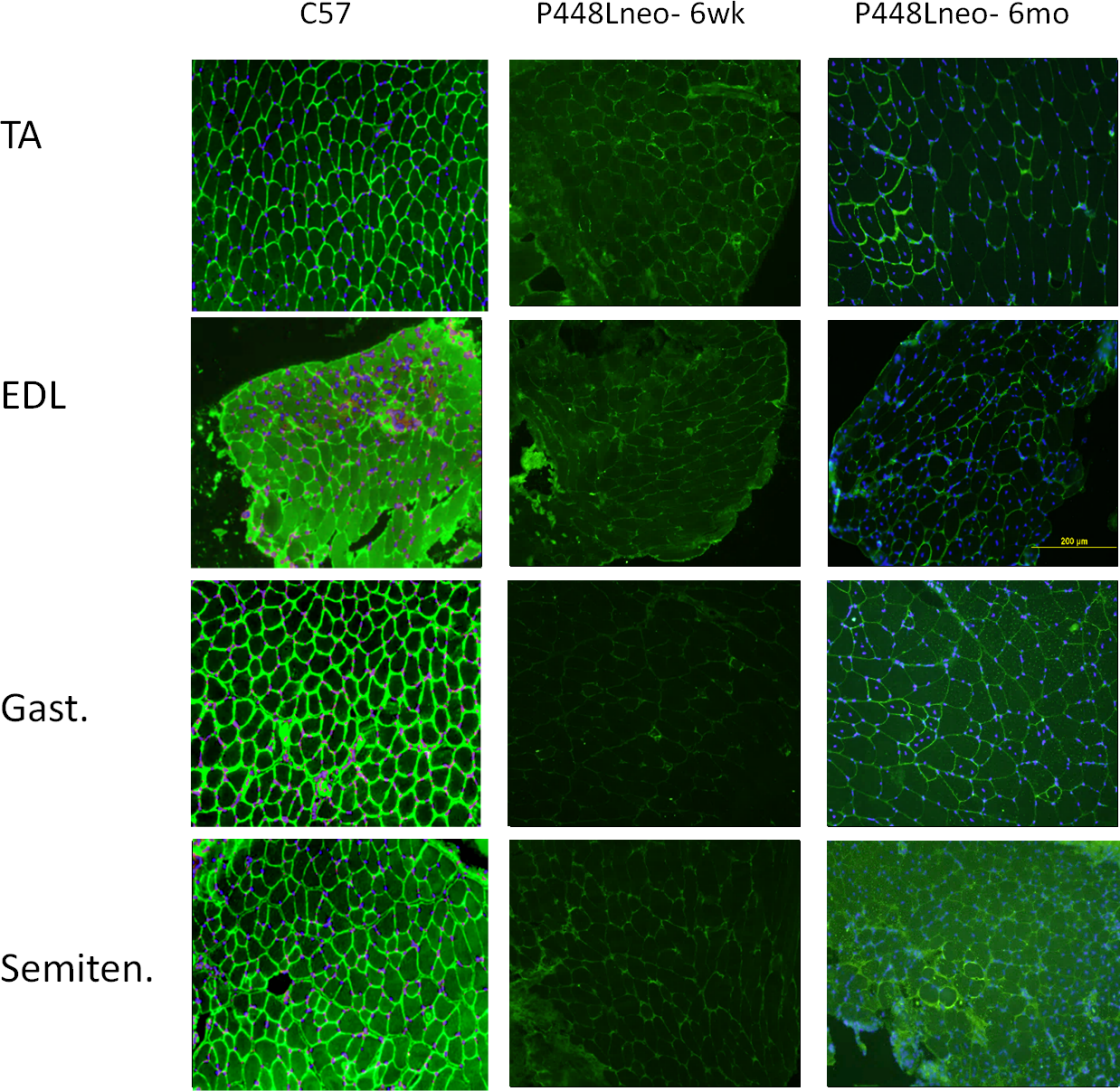
Presence of revertant fibers in hindlimb muscles of *P448Lneo-* mutant mouse. Tibialis anterior (TA), extensor digitorum longus (EDL), gastrocnemius (Gast.), and semitendinosus (Semiten.). Strong IIH6 staining shown in all muscle of C57, but almost absent in both 6 week and 6 month old P448Lneo-. Varying numbers of revertant fibers seen at the different ages of *P448Lneo-*. Images displayed at 20X magnification. Yellow bar (shown in 6 month EDL) represents 200 micron.

**Fig 2:**
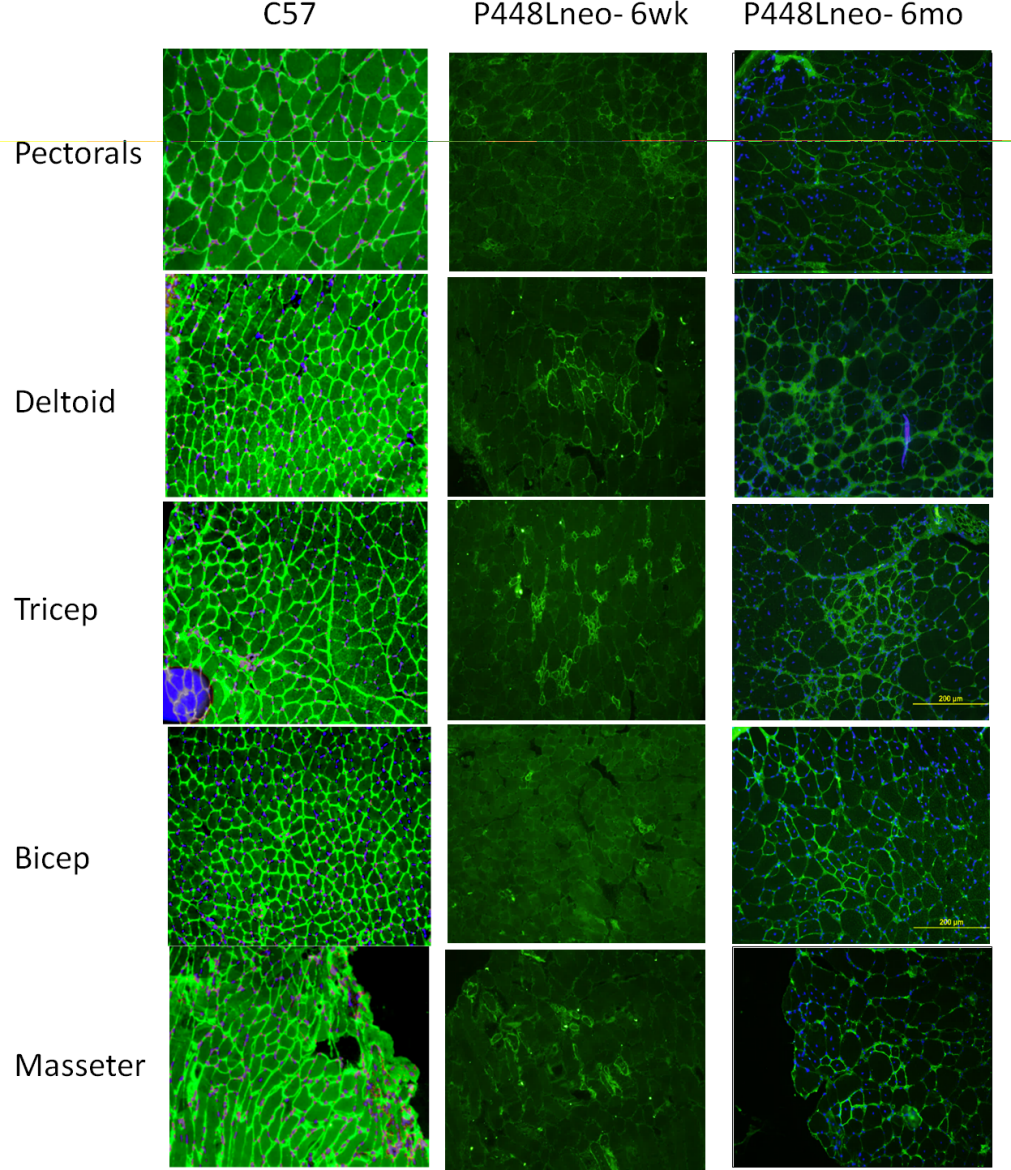
Presence of revertant fibers in forelimb, trunk, and head of *P448Lneo-* mutant mouse. Forelimb includes deltoid, triceps and bicep while trunk and head include pectorals and masseter respectively. IIH6 staining shown in all muscle of C57, but mostly absent in both 6 week and 6 month old P448Lneo-. Varying numbers of revertant fibers are seen at the different ages of *P448Lneo-*. Images displayed at 20X magnification. Yellow bar (shown in 6 month triceps) represents 200 micron.

**Fig 3:**
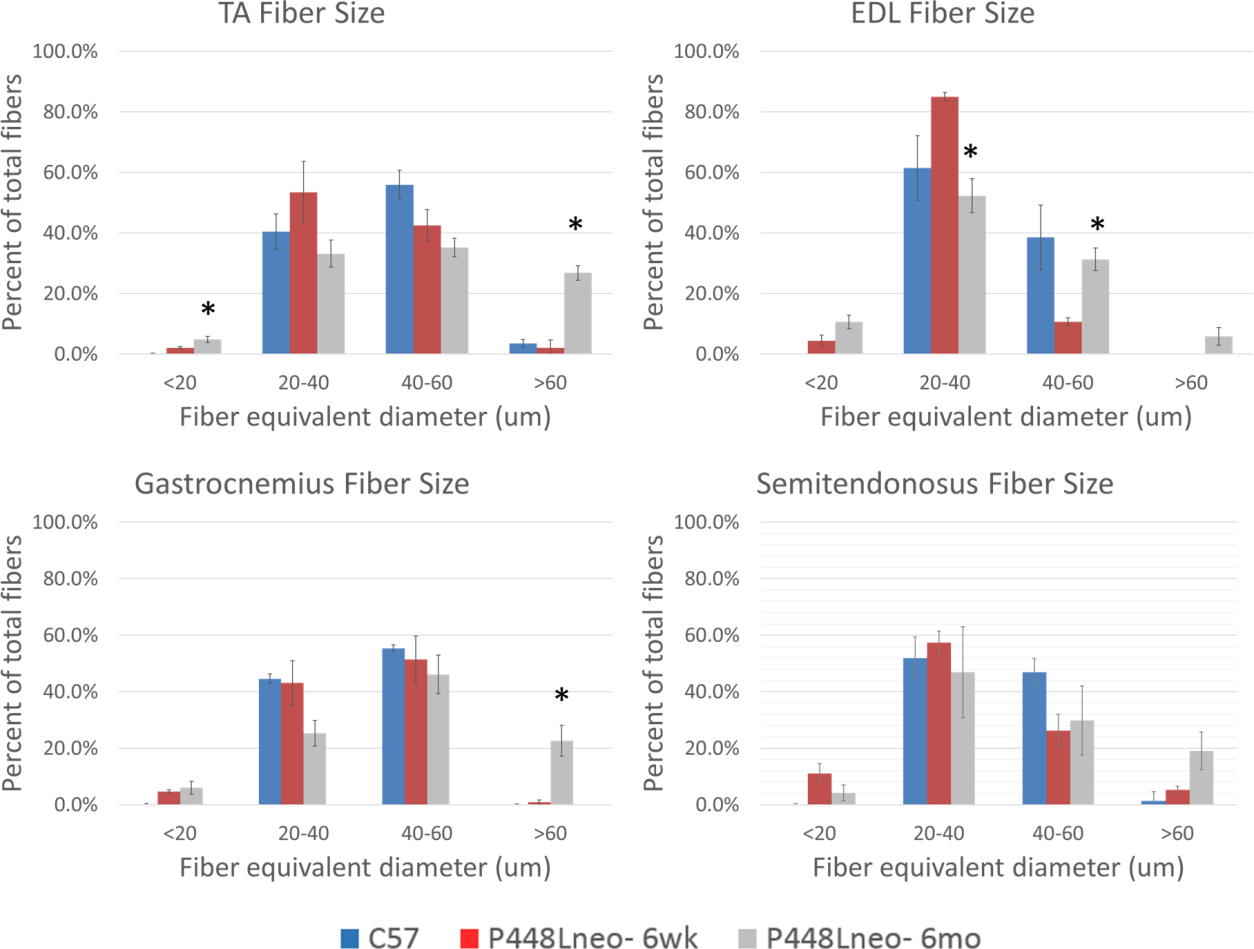
Increase in fiber size of hindlimb muscles of P448Lneo- mutant mouse. A clear increase in fiber size of the TA, EDL and gastrocnemius is seen from 6 weeks to 6 months of age with only a slight increase in the semitendinosus. However, a larger population of small fibers, <20 micron diameter, is seen in the EDL and gastrocnemius. Significant difference between 6 weeks and 6 months noted with *. Significance determined as p<0.05.

**Fig 4:**
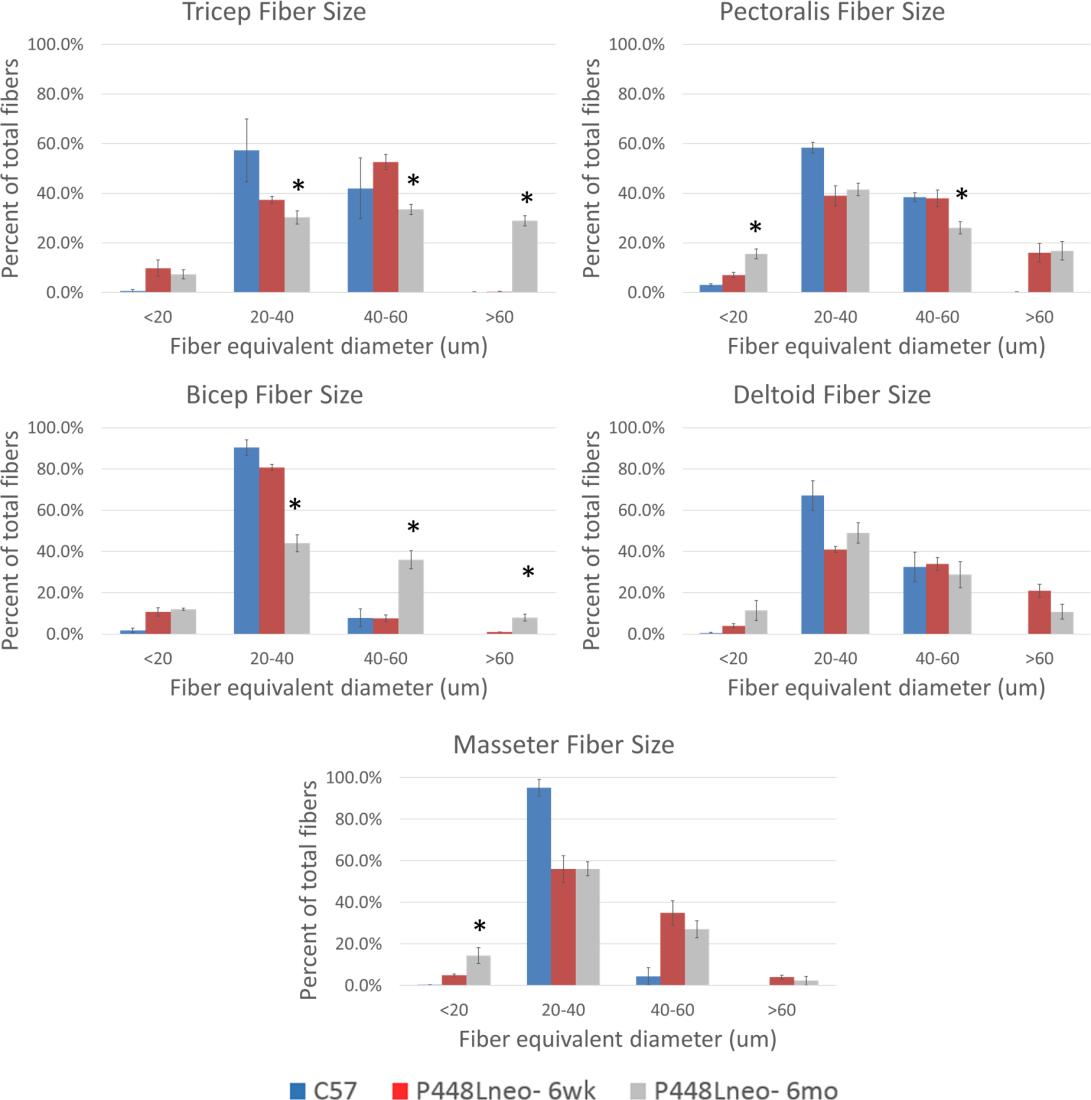
Fiber size of forelimb, trunk and head muscles of P448Lneo- mutant mouse. An increase in muscle fiber diameter is clear in both triceps and biceps with little to no increase in smaller diameter fibers. However, pectoralis, deltoid and masseter show an increase in fibers with small diameter, <20 micron. Significant difference between 6 weeks and 6 months noted with *. Significance determined as p<0.05.

We have demonstrated that restoration of F-α-DG in skeletal muscles of FKRP-P448L mutant muscles is closely associated with regeneration [24]. However, it is unclear if this applies to different muscles. We therefore examined a broader range of skeletal muscles to assess the potential variation between levels of regeneration and expression of F-α-DG. Triceps, biceps, deltoid, pectoralis, masseter, semitendinosus (semiten.) and extensor digitorum longus (EDL), as well as Tibialis anterior (TA) and gastrocnemius (gast.) of the P448Lneo- mutant mouse at 6 weeks and 6 months of age were examined with C57 as positive controls. Functional glycosylation of α-DG is largely absent in all examined skeletal muscle of the hind limb (Fig 1) as well as those in the forelimb, trunk and neck (Fig 2). However, revertant fibers are detected with varying frequency at both ages in all examined muscles (Table 1). To assess correlation between fiber regeneration and F-α-DG expression in the diseased muscles, the number of centrally nucleated fibers (CNF, representing the levels of regeneration) and IIH6 positive fibers (representing expression of F-α-DG) were counted. Revertant fibers, most of them small in size with central nucleation were identified in all muscles at the age of 6 weeks. The number of RF matched closely to that of CNF in most muscles, but variation was also clearly observed in the deltoid and biceps. (Table 1) This data is consistent to our early observation that regenerating fibers express F-a-DG during the first 2 weeks. But the significant variation between the two events suggest that muscle specific factors are also involved in the regulation of functional glycosylation. However, both CNF and revertant fibers were minimal in the EDL. The number of CNF increased significantly from 6 weeks to 6 months, with the triceps reaching as high as 72.9% of fibers. Concomitantly, the highest number of revertant fiber was also detected in the Triceps. In the EDL, the significant increase in revertant fibers was also correlated with the increase in CNF. These data further support the association between the two events. However, despite significant increase in the number of CNF in the Pectoral muscle from 6 weeks to 6 months, the number of revertant fibers dropped to only 6%, the lowest in all muscles (Table 1). Of note is the increase in fiber size as the disease progresses. This can be attributed to an increase in the population of hypertrophic fibers, especially in the triceps and biceps, but not in the pectoralis, deltoid or masseter (Figs 3 and 4). Also observed is an increased presence of muscle fibers of small size, <20 um diameter, in the EDL and gastrocnemius (Fig 3) as well as pectoralis, deltoid and masseter (Fig 4) which can be related to the general increase in CNF and RF as the disease progresses from 6 weeks to 6 months.

**Table 1:** Percentage of centranucleation (CN) and revertant fibers (RF) in 6 week (6WK) and 6 month (6MO) old *P448Lneo-* mice.

CN	6WK	6MO	RF	6WK	6MO
**Hindlimb**			**Hindlimb**		
TA	3.5%±0.4	***61*.*7%±3*.*4***	TA	3.7%±0.7	***23*.*5%±4*.*2***
EDL	1.2%±0.9	***51*.*2%±3*.*0***	EDL	0.9%±0.6	***17*.*5%±2*.*9***
Gastroc	3.9%±1.3	***39*.*4%±4*.*8***	Gastroc	4.7%±1.1	***17*.*6%±2*.*7***
Semiten.	5.1%±1.2	***37*.*2%±8*.*2***	Semiten.	2.2%±0.9	8.9%±3.4
**Forelimb**			**Forelimb**		
Triceps	11.5%±2.1	***69*.*1%±2*.*7***	Triceps	15.1%±3.6	***25*.*4%±3*.*7***
Biceps	5.6%±0.7	***51*.*8%±4*.*9***	Biceps	8.4.0%±2.5	16.7%±5.7
Deltoid	15.4%±2.6	***52*.*9%±2*.*5***	Deltoid	3.8%±0.7	***14*.*2%±2*.*6***
**Trunk**			**Trunk**		
Pectorals	17.1%±3.1	***62*.*5%±2*.*6***	Pectorals	12.8%±3.3	***6*.*2%±1*.*2***
**Head/Neck**			**Head/Neck**		
Masseter	7.5%±1.4	***33*.*1%±1*.*5***	Masseter	7.1%±2.1	***13*.*6%±2*.*3***

A gross increase is seen in centranucleation of 6 month old muscle fibers. An increase is seen in the presence of revertant fibers in all muscle except pectorals which show a decrease from 6 weeks to 6 months of age. Percentage of total expressed as mean ± SEM. Statistical significance is determined by t-test with p<0.05. Significance indicated in bold and italicized values.

### F-α-DG in peripheral nerve

**Fig 5:**
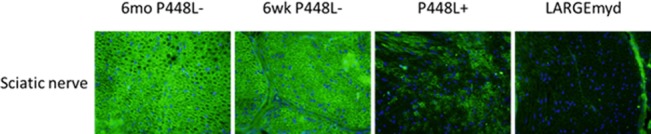
F-α-DG in sciatic nerve. Strong IIH6 staining is demonstrated in the sciatic nerve of 6 week and 6 month old P448Lneo- with patchy expression in the P448Lneo+. Expression surrounding the axons is absent in the LARGE^myd^. Images are taken at 20X magnification.

**Fig 6:**
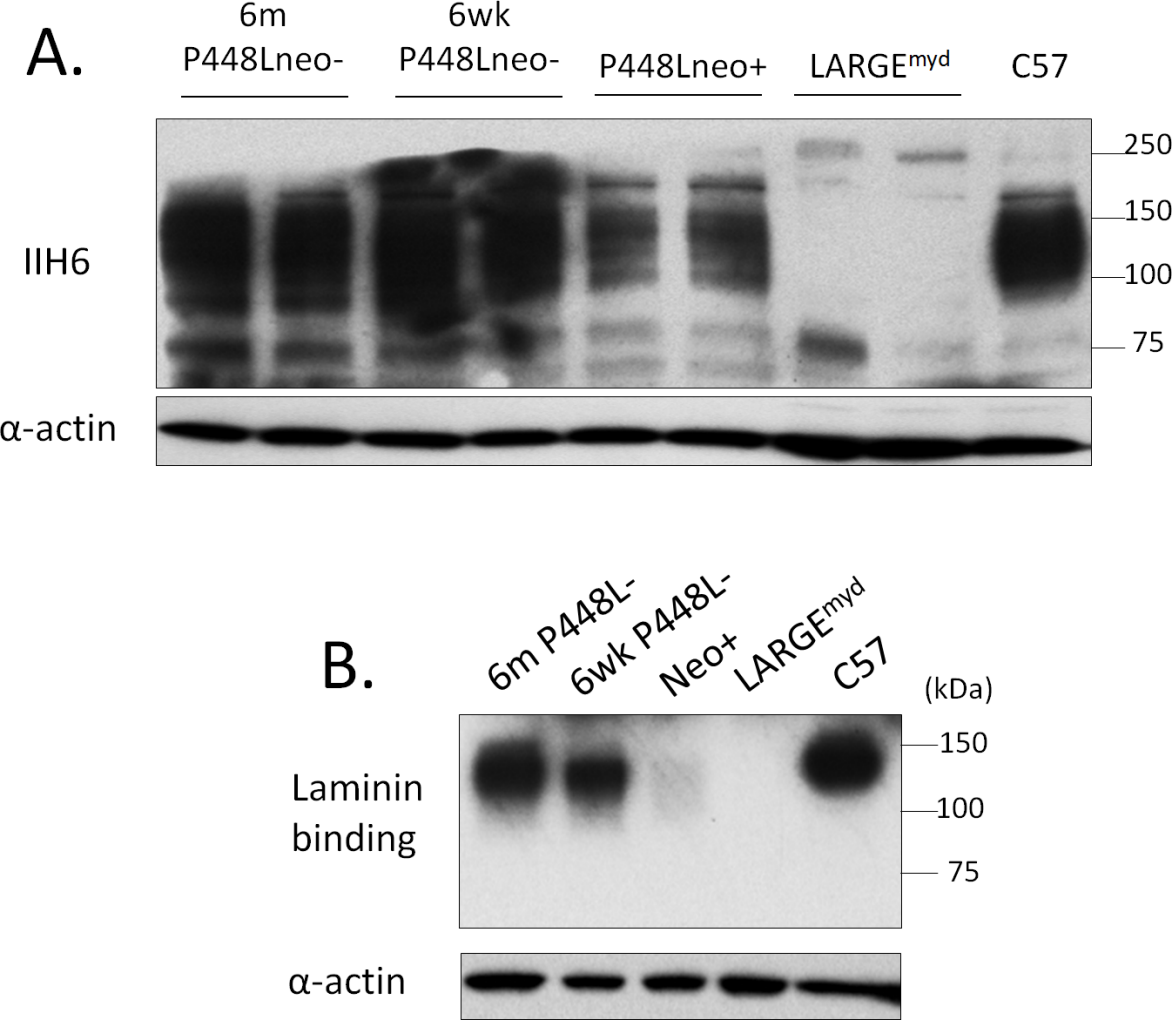
Western blot detection of functionally glycosylated α-DG in sciatic nerve. A. Strong expression is seen in both the 6 week and 6 month old P448Lneo- mouse comparable to C57 control tissue. A decreased expression is seen in the Neo+ and absence in the LARGE^myd^. B. Laminin overlay demonstrated functional binding of α-DG to laminin in the sciatic nerve. There is minimal binding in the P448Lneo+ with absence in the LARGE^myd^.

In peripheral nerve, F-α-DG is expressed in Schwann cells of normal mice. F-α-DG is lost in both *Fukutin* mutant and *LARGE*^*myd*^ mice [28]. The terminal nerves within the muscle tissue of the P448Lneo- mutant mice stained positively with the IIH6 antibody whereas those in the muscle of the *LARGE*^*myd*^ mice were negative (S1 Fig), suggesting that the P448Lneo- mutant FKRP could be sufficient for functional glycosylation of α-DG in the peripheral nerve. However, the background of mouse tissue with monoclonal antibody and the small volume of the nerve tissue make it difficult to confirm the expression. To determine if the peripheral nerve Schwann cells with FKRP P448Lneo- mutation retains the functionally glycosylated α-DG we dissected sciatic nerve of 6 week old C57, P448Lneo-, P448Lneo+ and LARGE^myd^ for immunohistochemistry and western blot. Similar to previous reports, sciatic nerve of C57 mice stained strongly with IIH6 and ring-shaped signals around the nerve axon, consistent with the expression of the α-DG in Schwann cells. Surprisingly, 6 week old P448Lneo- sciatic nerve showed strong IIH6 signal, with the intensity equivalent to that in the C57 controls. Further examination of the 6 month old P448Lneo- showed the same pattern and intensity (Fig 5). The sciatic nerve of P448Lneo+ mice demonstrated clear but patchy staining around a proportion of the nerve axons. The intensity of the signal was obviously weaker than that observed in both P448Lneo- and normal C57 mice. In contrast, IIH6 staining was completely absence in LARGE^myd^ nerve. This is consistent with western blot analysis which showed near normal levels of F-α-DG, as demonstrated by IIH6 binding, in both 6 week and 6 month old P448Lneo-, reduced levels in P448Lneo+, and loss in LARGE^myd^ (Fig 6). Consistently, Laminin overlay showed positive binding of the P448Lneo- mice with signal intensity similar to that of C57 controls. A weak band was detected with IIH6 in the P448Lneo+ mice, but absent in the LARGE^myd^ (Fig 6). The molecular size of the IIH6 recognized F-α-DG in the nerves of both normal and P448L mutant mice was the same, about 120kDa, smaller than that (140-200kDa) in skeletal muscles, but similar to that in brain (S2 Fig). This is consistent with a previous study demonstrating that LARGE is required for the glycosylation of α-DG in the peripheral nerves [29].

### F-α-DG in submandibular salivary gland

**Fig 7:**
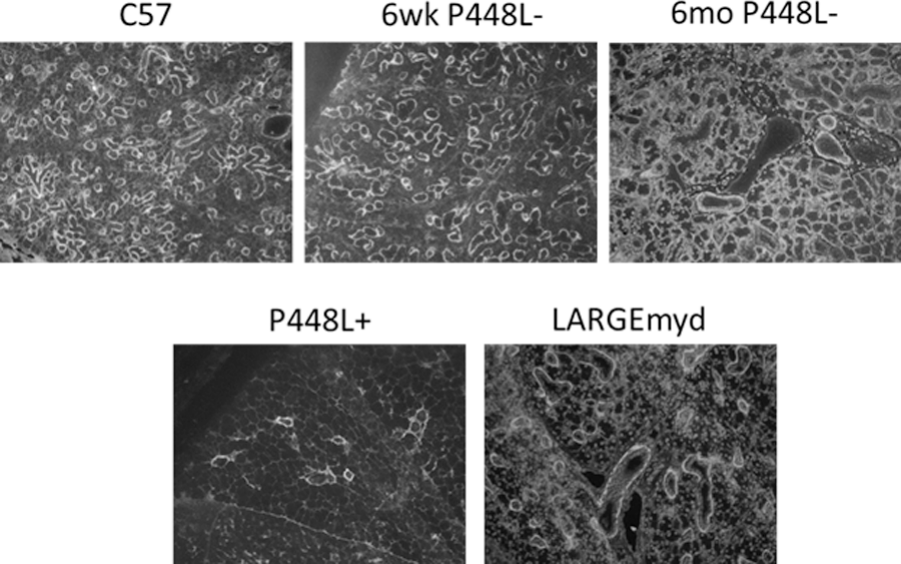
F-α-DG in the salivary gland. Strong IIH6 staining is seen around the striated ducts and acini of C57, P448L- (both 6 weeks and 6 months of age) as well as the LARGE^myd^. However, detectable IIH6 staining is seen around the striated ducts of P448L+ and not acini. Images are 20X magnification.

**Fig 8:**
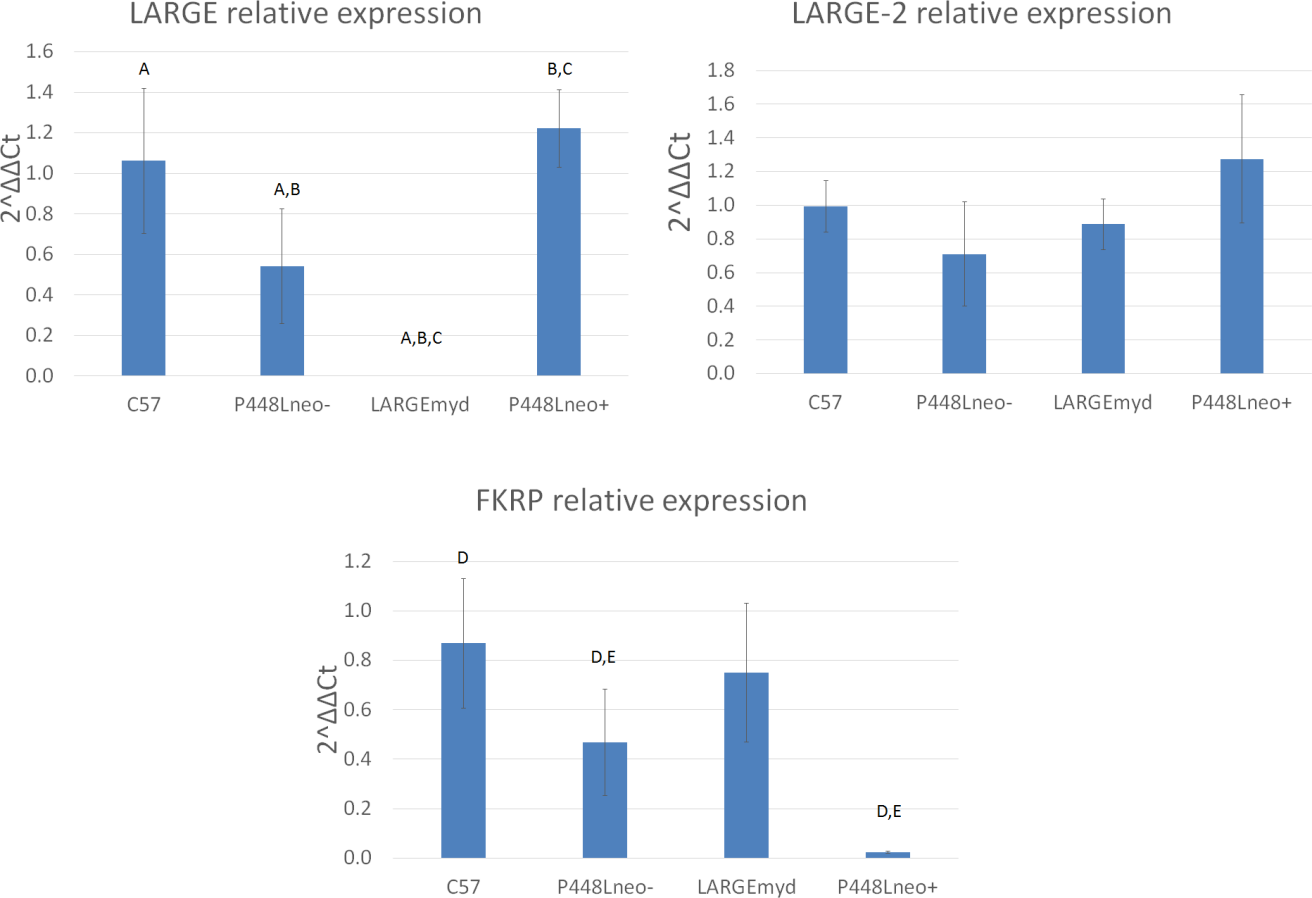
Quantitative real time-PCR of salivary gland. *FKRP* expression levels are normal in LARGE^myd^ compared to C57 controls while significantly reduced levels are seen in the P448Lneo- and almost absent in the P448Lneo+. *LARGE* expression levels are high in the C57 as well as both P448Lneo- and neo+, although a decrease is noted in the P448Lneo-. However, as expected, LARGE expression levels are absent in the LARGE^myd^ mouse. *LARGE-2* expression shows near normal levels for all three mutant mouse models with no significant difference for any. Significant differences are noted with the same letter as determined by t-test with a p<0.05.

Early studies have established the fact that epithelium, including salivary glands, also express F-α-DG. Interestingly, this expression in salivary glands was reported to not be LARGE dependent as normal expression is maintained in LARGE^myd^ mice. We therefore examined the submandibular salivary gland of the FKRP mutant mice to assess the role FKRP plays in the functional glycosylation of α-DG. F-α-DG was expressed in the normal C57 mouse with strongest signal in the duct cells and only faint staining at the basal site of the acini. Similar to the normal C57, the P448Lneo- mouse shows high levels of IIH6 signal surrounding the basal membrane of the striated ducts, and weak staining was also detected around the acini. However, in the P448Lneo+, expression of F-α-DG was observed strongly only surrounding the striated ducts and signals were hardly detectable in other epithelial cells of the gland. Of note is the submandibular salivary glands of LARGE^myd^ mice. In contrast to the nerve, near normal expression is demonstrated surrounding the striated ducts and acini (Fig 7). Similar to the expression in skeletal muscles, Quantitative real-time PCR analysis showed slightly reduced levels of *FKRP* in P448Lneo- mutant mice and a near loss of expression in P448Lneo+ mice. *FKRP* levels are normal in LARGE^myd^ mice. As expected, *LARGE* expression in both P448Lneo- and P448Lneo+ mice were similar to the normal C57, but absent in LARGE^myd^ mice. Quantitative real time-PCR showed similar levels of *LARGE-2* in all four mouse types (Fig 8) consistent with earlier reports of higher levels of LARGE-2 in salivary gland [30, 31]. Taken together, the results suggest that *FKRP* expression is critical for both LARGE and LARGE-2 mediated F-α-DG in the salivary gland, but only limited levels of FKRP, or even mutant FKRP could be sufficient for normal levels of F-α-DG. The results also support the earlier indication that LARGE-2 is sufficient for the functional glycosylation of α-DG in the salivary gland.

### F-α-DG in large intestine

**Fig 9:**
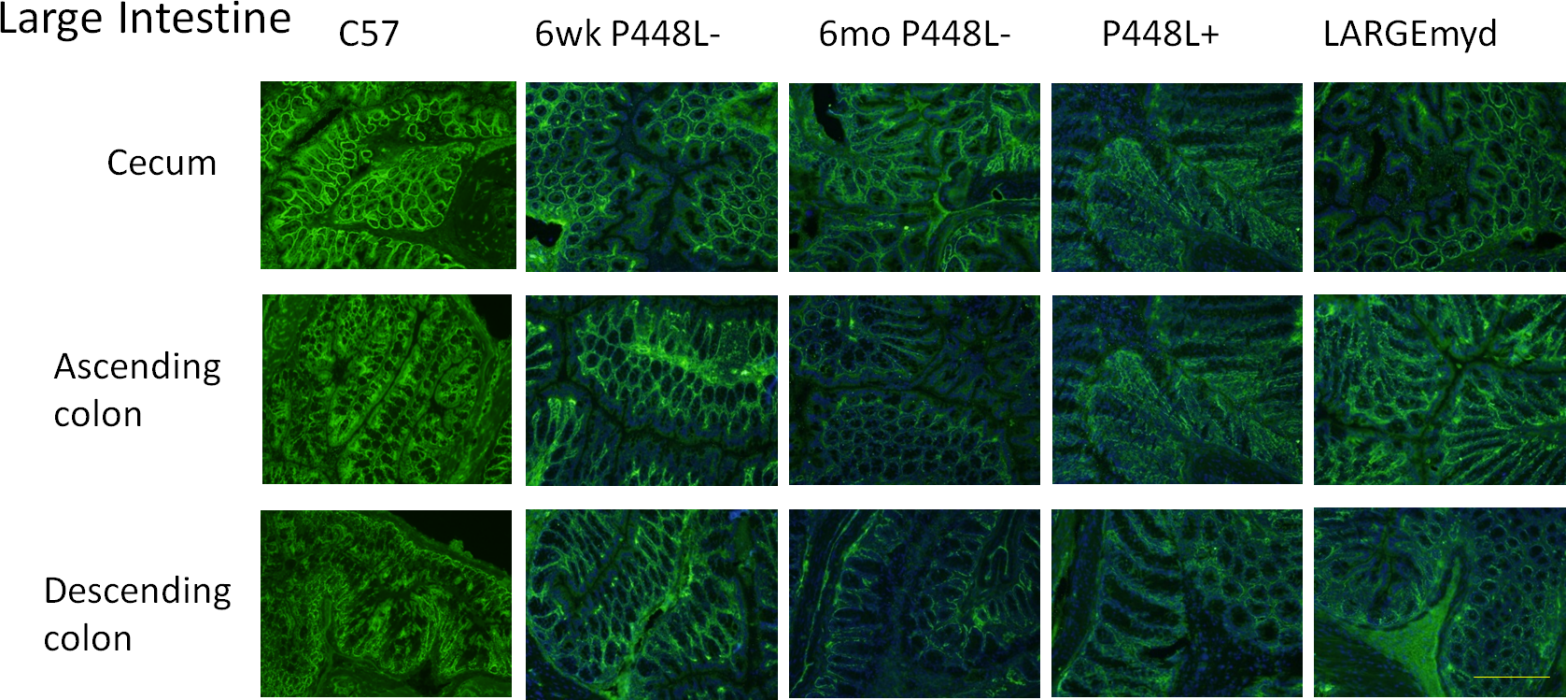
Expression of F-α-DG in regions of large intestine. Strong expression is demonstrated in the epithelial cells in P448Lneo- mutant mouse with patchy expression in the P448Lneo+ and LARGE^myd^ mouse.

**Fig 10:**
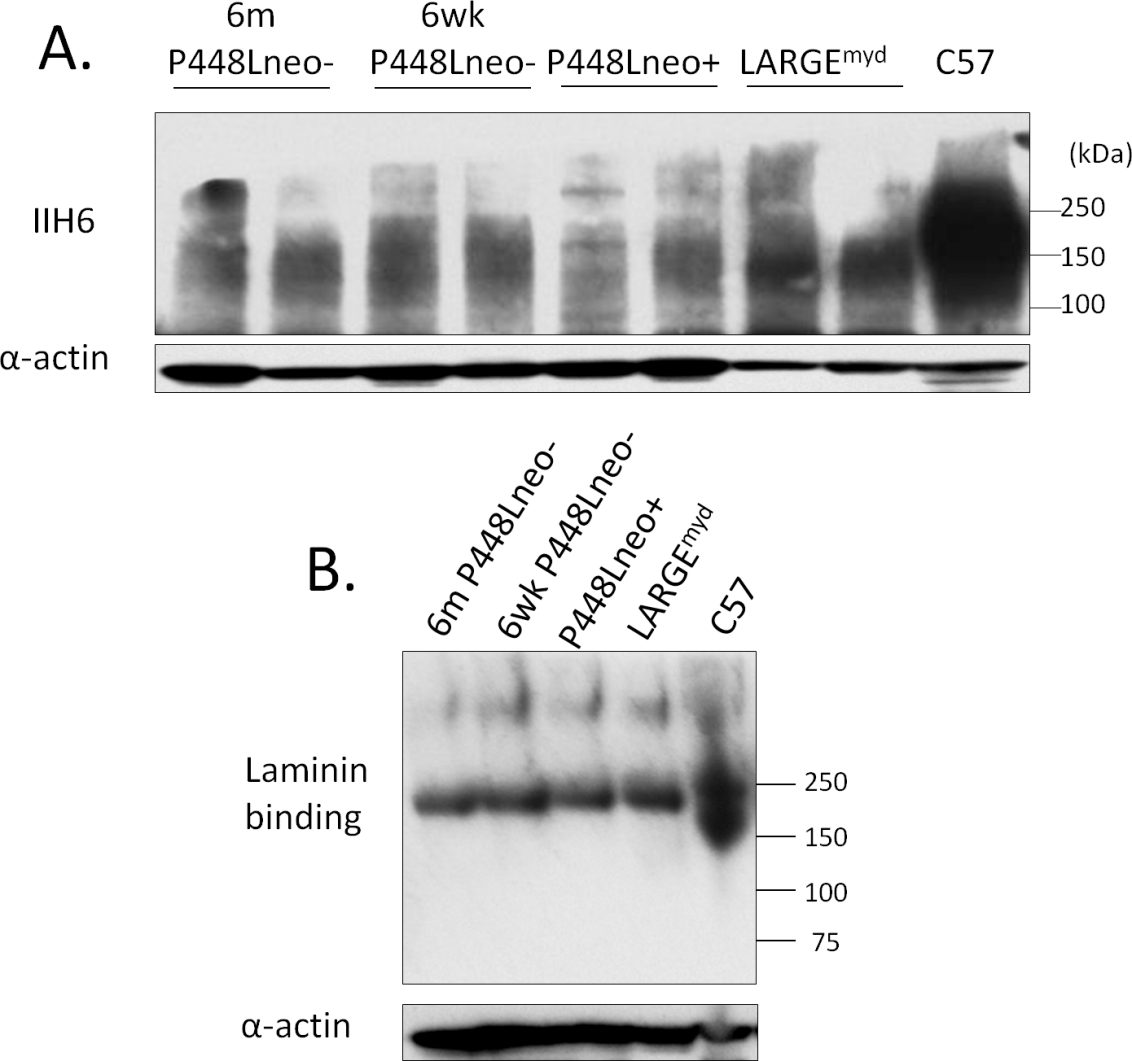
Western blot detection of functionally glycosylated α-DG in cecum. A. Although lower than C57 control tissue, the expression of IIH6 is clearly visible in the P448Lneo- and P448Lneo+ as well as LARGE^myd^. However, these levels appear to be higher in the LARGE^myd^ compared to both P448L mutant mice with lower expression in the P448Lneo+. B. Functional binding of the α-DG to laminin is demonstrated in the cecum of all animals although in reduced levels in the mutant mice compared to C57 controls.

The presence of dystroglycan has been previously noted in normal mouse intestine[32]. Durbeej et al., using antibodies directed toward both alpha- and beta-dystroglycan, demonstrated expression in the basement membrane side of the epithelial cells of intestinal glands as well as the epithelial cell surfaces. However, expression was limited to the basement membrane side in the villi. Furthermore, α-DG expression was absent in the intestinal epithelium of patients with FCMD [33]. Similar to these earlier studies, large intestine from control C57 mice shows strong expression of F-α-DG in the basement membrane sides of epithelium, especially the glandular epithelium. However, this expression is reduced in both the P448Lneo- and LARGE^myd^ mouse and greatly reduced in the P448Lneo+ (Fig 9). These results were confirmed in western blot analysis showing reduced levels in the mutant mouse as well as positive laminin overlay although in reduced levels compared to C57 controls (Fig 10).

## Discussion

The presence of revertant fibers has been demonstrated in both human and mouse models [25, 34, 35]. These fibers are generally small in size, clustered in small groups, and exhibit near normal levels of F-α-DG. Previous data from our laboratory and others have suggested that these fibers are regenerating fibers [24, 36]. Although the mechanism(s) involved in the restoration of strong F-α-DG are largely unknown, this feature could be useful for understanding the status of the diseased muscles. In dystrophic muscles of mouse models, progression of the disease is known to be indicated by a significant increase in centrally nucleated fibers. In this study, we show that increase in revertant fibers expressing F-α-DG could be considered as a sign of continuous muscle degeneration and regeneration. Our previous study showed that F-α-DG is expressed strongly in the newly regenerated myotubes and remains detectable for only about 4 weeks. It is therefore likely that reduced numbers of revertant fibers, in strains of mice which maintain a good regenerative capacity till very late of their life span, indicate a reduction in muscle regeneration, thus degeneration in a recent past. This is clearly different from central nucleation which could last for many months in dystrophic mouse muscles [37], making it difficult to assess the recent status of muscle regeneration by percent of CNF. However, the presence and number of revertant fibers are variable from muscle to muscle and age-related with older mice having reduced regenerative capacity, thus only same muscle of same aged groups can be comparable. The significance of the number of revertant fibers in clinics is not understood and the results from the current study may not be applicable to all dystroglycanopathies. For example, it is clear from animal model of mild LGMD2I with L276I mutations, a large proportion of the muscle fibers including those mature fibers without central nucleation, express variable levels of F-α-DG. Distinguishing these background F-α-DG positive fibers from newly regenerated fibers will be difficult. However, reports from clinic biopsy sample analyses indicate that only a small proportion of muscle fibers from a majority of LGMD2I patients express F-α-DG [22]. This pattern is therefore similar to that observed in the P448L mutant mouse muscles, supporting the view that the revertant fibers in patient muscles represent the presence of regeneration.

Interestingly, the presence of centrally nucleated and revertant fibers differs by muscle and at different time points. Such variation has several implications. First, while most muscles already show a considerable number of muscle fibers with centranucleation and expression of F-α-DG, EDL muscle mostly lacks CNF and RFs. However, the number of both markers becomes similarly high at the age of 6 month, suggesting that degeneration and regeneration occurs later in EDL than other muscles. This is clearly different from the dystrophic mdx mice which have been reported with pathological changes as early as 3 weeks [38] suggesting that defects in different dystrophin-dystroglycan-complex component affect muscles differentially. Second, while the TA, EDL and Gastrocnemius show a large and parallel increase in both centrally nucleated and revertant fibers from 6 weeks to 6 months of age, the significant increase in the number of CNF of pectoralis is associated with a reduction in the number of revertant fibers during the same time frame. The mechanism(s) involved is not understood, but one possible explanation would be that the regeneration rate and central nucleation varies greatly in different muscle and in different time frame. It is also possible that other factor(s) affecting F-α-DG exists in different muscles. Also important is the fact that the number of RF is nearly always lower than that of CNF further support our hypothesis that muscle regeneration is the primary factor for revertant expression of F-a-DG. Furthermore, understanding the pattern of variation in expression of F-α-DG in different muscles would be important for clinical trials aiming to restore F-α-DG. It is expected that any specific experimental therapy will also have variable levels of effect on different muscles. An example is AAV-mediated gene replacement therapy which induce variable levels of transgene expression and consequently the levels of F-α-DG in different muscles. Equipped with both background and therapeutic levels of marker gene expression would allow us to determine which muscle might be more relevant and reliable for assessing efficacy of specific therapeutic strategy.

The P448Lneo- mouse displays a primarily myopathic phenotype with little to no changes in the eye or brain. However, the addition of the Neomycin cassette (P448Lneo+) exasperates the disease and produces animals with clear brain and eye defects [25, 27]. The underlying mechanism(s) for this difference is unknown but it has been linked to the levels of mutant FKRP expression, which is greatly reduced in the neo+ model compared to the neo- (Fig 8) [25]. Consistently, revertant fibers are only observed in the skeletal muscles of P448Lneo- mice, but not in the P448Lneo+. Furthermore, expression of near normal levels of F-α-DG is observed in the regenerating skeletal muscles and now in the sciatic nerve of P448Lneo- mouse, but none or only patchy staining in P448Lneo+. This, together indicates that FKRP essential for the glycosylation of α-DG in the nervous system. While currently there is no reliable mean for detecting the levels of FKRP protein in the nerve itself, the difference in levels of partially-functional FKRP (as reported by [23, 24]) likely also explain the glycosylation patterns in the sciatic nerve of these two animal models. The near normal IIH6 expression in the peripheral nerve of P448Lneo- mutant mice further support the notion that a majority of mutant FKRPs with milder clinic manifestation than those with P488L mutation likely retains sufficient residual function for near normal levels of F-α-DG in peripheral nerves. Similar to muscle, the loss of LARGE affects the functional glycosylation resulting in a complete lack of IIH6 staining in the sciatic nerve. This supports the previous observation that loss of F-α-DG is responsible for peripheral nervous system abnormalities [8] including impaired nerve conductance, altered axonal radial sorting, and impaired recruitment of several neuromuscular junction components [39].

The levels of IIH6 positive F-α-DG in the salivary gland varies significantly in the models examined, being highest in the P448Lneo-, patchy to no expression in the P448Lneo+, but near normal expression in the LARGE^myd^ mouse. Our results of qRT-PCR for detection of *LARGE* and *LARGE-2* provides further evidence supporting the earlier conclusion that *LARGE-2* expression is responsible for the normal levels of F-α-DG in salivary glands of the LARGE^myd^ mice. However, the biological function of LARGE-2 also depends on the function of FKRP as the levels of F-α-DG is significantly lower in the P448Lneo+ than that in the P448Lneo- mice. Interestingly, the expression of the F-α-DG in the large intestine follows a similar pattern as in the salivary gland, with considerably higher levels in both P448Lneo- and LARGE^myd^ mice than that in the P448Lneo+. This can be explained by the fact that *LARGE-2* is expressed, in large intestine although at levels lower than that in salivary glands [31]. Also important is that the level of LARGE2 is normally higher than LARGE in large intestine. Thus, F-α-DG in large intestine is likely mediated by both LARGEs and lack of *LARGE* expression reduces the level of F-α-DG, but the presence of LARGE2 maintains great portion of the functional glycosylation. These results support that LARGE 2 plays major role in the F-a-DG in many different epithelial cells as indicated by poly A dot-blot tests [31].

Taken together our data demonstrates that FKRP functions are essential for the glycosylation of α-DG and mutant FKRP, associated with severe CMD in clinics, remains partially functional in all tissues. Expression of near normal levels of F-α-DG in FKRP mutant animals is tissue dependent and can be modulated by the process of regeneration and maturation [23, 24]. While LARGE plays a major role in the glycosylation of α-DG in muscles and nerve systems, LARGE-2 appears more critical for epithelial tissue, such as salivary gland and large intestine. Further study of glycosylation in these different tissues might enable us to identify factors other than *LARGE* and *FKRP* modulating the efficiency of functional glycosylation of α-DG.

## Supporting information

S1 Fig IIH6 staining of forearm. IIH6 staining of cross section of the forearm of 6 week (A) and 6 month (B) P448Lneo- mutant mouse. IIH6 staining is absent from muscle fibers (white star) however strong and patchy expression is seen in 6 week and 6 month old nerve, respectively (white arrow). Image is cropped from 20X magnification.
(DOCX)

S2 Fig Comparison of molecular weight (kDa) of glycosylated a-DG in different tissues of wild type C57. The molecular weight of the sciatic nerve (SN) is smaller than that of skeletal muscle, at ~150 kDa, as opposed to ~200kDa for skeletal muscle (represented by the quad). This molecular weight matches more closely to that of the brain.
(DOCX)
