# Supplementary material for: Distinct expression of functionally glycosylated alpha-dystroglycan in muscle and non-muscle tissues of FKRP mutant mice
Source: PLoS One. 2018 Jan 10;13(1):e0191016. doi: 10.1371/journal.pone.0191016 (PMC5761899; doi:10.1371/journal.pone.0191016)
Supplement: S1 Fig — IIH6 staining of cross section of the forearm of 6 week (A) and 6 month (B) P448Lneo- mutant mouse. IIH6 staining is absent from muscle fibers (white star) however strong and patchy expression is seen in 6 week and 6 month old nerve, respectively (white arrow). Image is cropped from 20X magnification. (DOCX) [file pone.0191016.s001.docx]

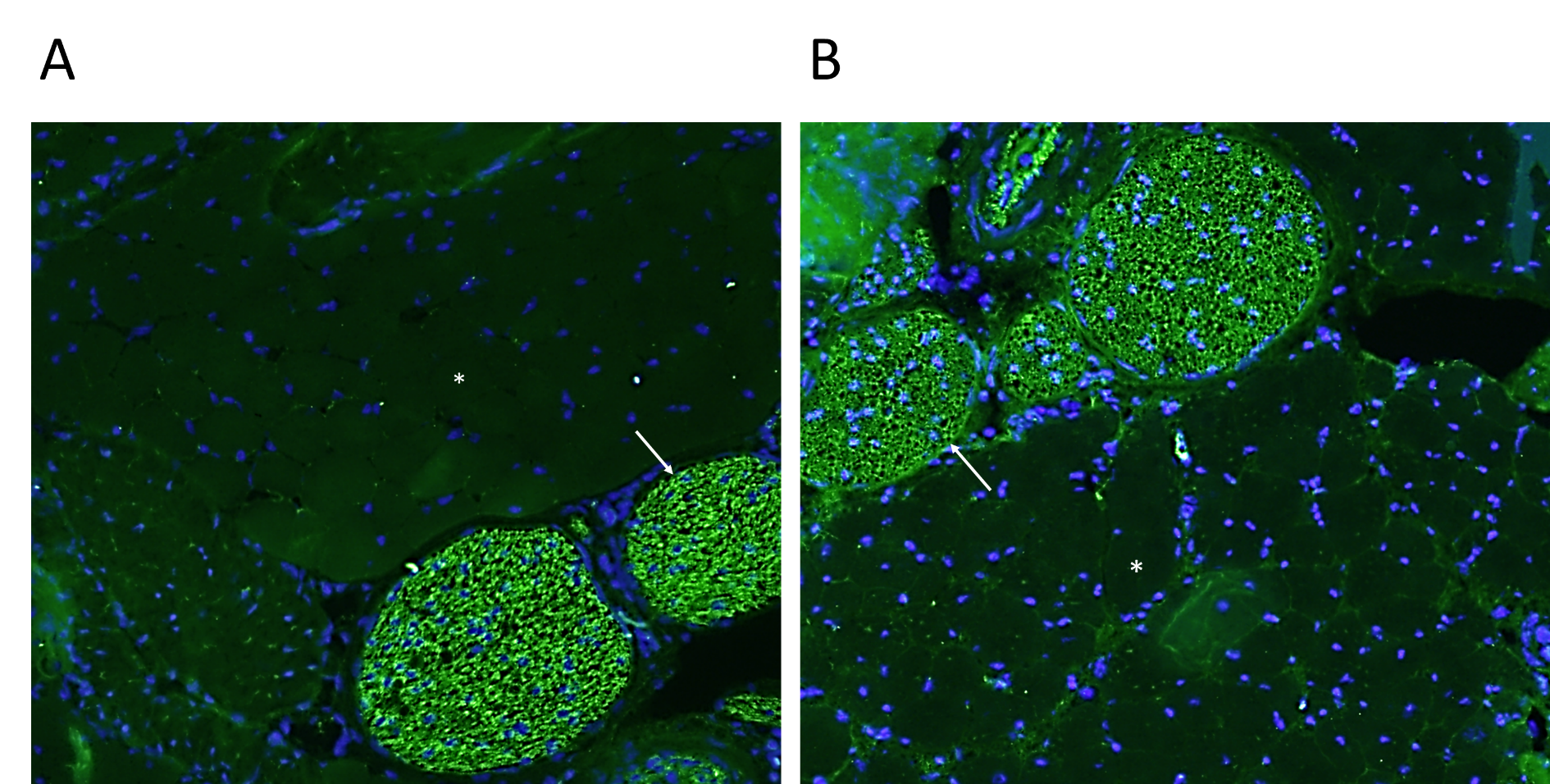


**S1 Fig. IIH6 staining of forearm**. IIH6 staining of cross section of the forearm of 6 week (A) and 6 month (B) P448Lneo- mutant mouse. IIH6 staining is absent from muscle fibers (white star) however strong and patchy expression is seen in 6 week and 6 month old nerve, respectively (white arrow). Image is cropped from 20X magnification.
