# Supplementary material for: Distinct expression of functionally glycosylated alpha-dystroglycan in muscle and non-muscle tissues of FKRP mutant mice
Source: PLoS One. 2018 Jan 10;13(1):e0191016. doi: 10.1371/journal.pone.0191016 (PMC5761899; doi:10.1371/journal.pone.0191016)
Supplement: S2 Fig — The molecular weight of the sciatic nerve (SN) is smaller than that of skeletal muscle, at ~150 kDa, as opposed to ~200kDa for skeletal muscle (represented by the quad). This molecular weight matches more closely to that of the brain. (DOCX) [file pone.0191016.s002.docx]

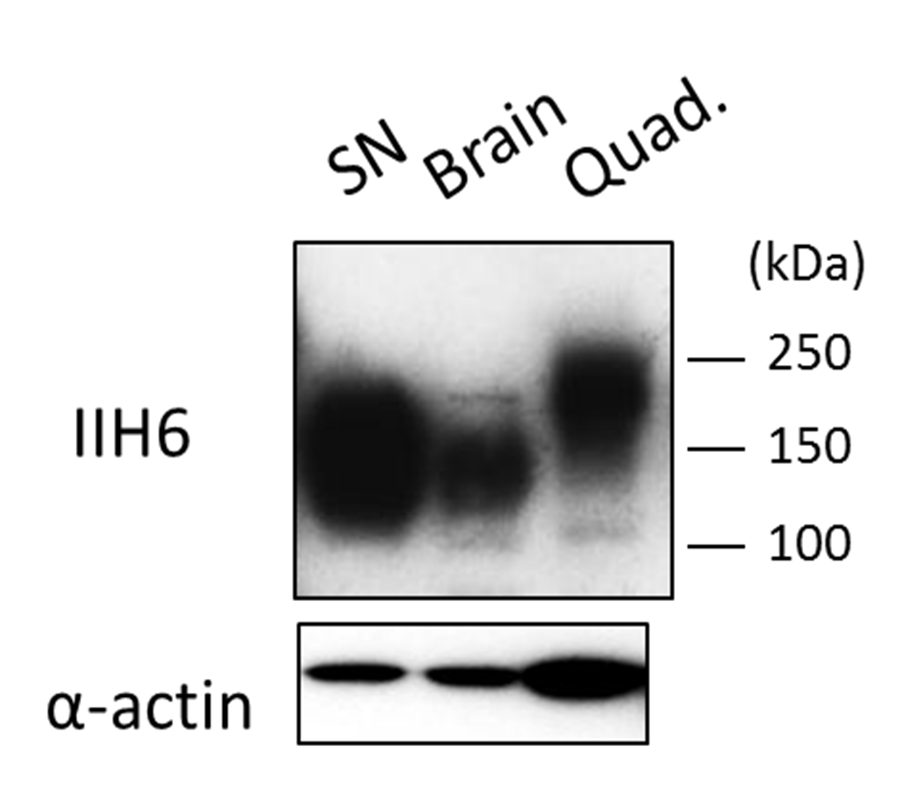


**S2 Fig. Comparison of molecular weight (kDa) of glycosylated a-DG in different tissues of wild type C57**. The molecular weight of the sciatic nerve (SN) is smaller than that of skeletal muscle, at ~150 kDa, as opposed to ~200kDa for skeletal muscle (represented by the quad). This molecular weight matches more closely to that of the brain.
